# Supplementary material for: Individual differences in fear acquisition: multivariate analyses of different emotional negativity scales, physiological responding, subjective measures, and neural activation
Source: Sci Rep. 2020 Sep 17;10:15283. doi: 10.1038/s41598-020-72007-5 (PMC7498611; doi:10.1038/s41598-020-72007-5)
Supplement: Supplementary file 1 — Supplementary file1 [file 41598_2020_72007_MOESM1_ESM.docx]

Supplementary Information to:

**Individual differences in fear acquisition: Multivariate analyses of different Emotional Negativity scales, physiological responding, subjective measures, and neural activation**

Rachel Sjouwerman, Robert Scharfenort & Tina B. Lonsdorf

**Exploratory analyses testing for an association between STAI-T scores and awareness**

**Study 1: Testing for an association with the STAI-T score and US intensity as well as awareness**

As previous research has suggested an association between awareness and US intensity with trait-anxiety ^for a review see 1^ exploratory analyses were conducted for Study 1 to exclude that results are biased by these factors. In brief, neither awareness of CS contingencies nor US intensity were significantly associated with the STAI-T: US intensity in mA did not correlate with STAI-T scores (r = -0.06, p = 0.26). Individuals aware (n = 236, mean STAI-T = 38) unaware (n = 87, mean STAI-T = 40) or uncertain (n = 21, mean STAI-T = 39) of CS-US contingencies did not differ significantly in STAI-T scores (F(3,297) = 1.51, p = 0.21).

**Study 2: Testing for an association with the STAI-T score and US intensity as well as awareness**

Finally, as previous research has suggested an association between awareness and US intensity with trait-anxiety ^for a review see 1^ exploratory analyses were conducted for study 2 to exclude that results are biased by these factors. In brief, neither awareness of CS contingencies nor US intensity were significantly associated with the STAI-T: US intensity in mA did not correlate with STAI-T scores (r = -0.11, p = 0.24). Individuals aware (n = 101, mean STAI-T = 35) and unaware (n = 12, mean STAI-T = 32) of CS-US contingencies did not differ significantly in STAI-T scores (Welch two sample t-test: t(20.5) = 1.64, p = 0.16).


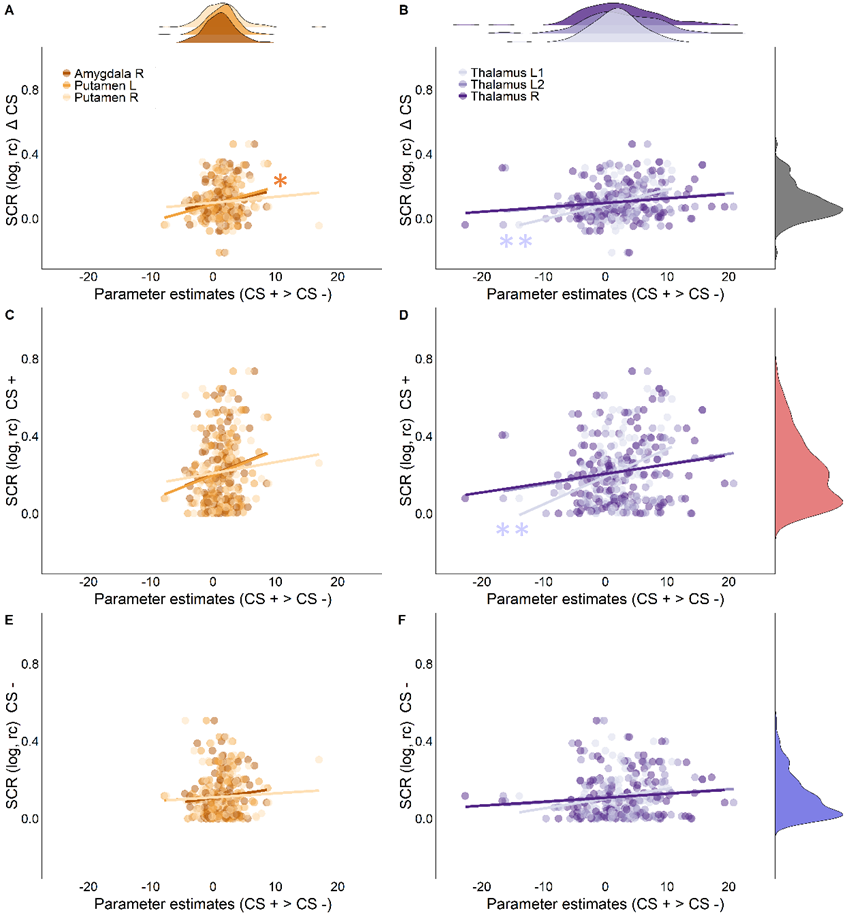


**Supplementary Figure 1**. Scatterplots serving illustrative purposes for (A,B) CS+/CS- discrimination (C,D) CS+, and (E,F) CS- in SCR responding and parameter estimates for CS + > CS – contrasts in the six brain regions significantly associated with the STAI-T (peak voxel parameter estimates extracted from individual first levels). The left panel shows the (A,C,E) right amygdala, left and right putamen and the right panel shows (B, D, E) two clusters in the left thalamus, and the right thalamus. Density distributions are shown on top of the figure for the parameter estimates (regions are color coded). Densities on the right side of each plot show SCR for CS discrimination, CS+ responding, and CS- responding respectively. * indicates p_BH_ < 0.05, ** indicates p_BH_ < 0.01.

**Supplementary references**

1. Lonsdorf, T. B. & Merz, C. J., More than just noise: Inter-individual differences in fear acquisition, extinction and return of fear in humans - Biological, experiential, temperamental factors, and methodological pitfalls. *Neuroscience and Biobehavioral Reviews* **80**, 703–728 (2017).
